# Making Coffee

**Published:** 1871-02

**Authors:** 


### MAKING COFFEE.

Make a bag of felt or heavy woolen flannel long enough to reach from the top to the bottom of the coffee-pot, with a wire attached to keep the bag upright, put the fresh ground coffee in the bag, pour on boiling water, and it is at once fit for use; the water takes the strength out of the coffee and passes through the flannel clear with all its aroma.

Americans persist in boiling the coffee, thus driving away its most delicious quality.

The French put the ground coffee in a tin cup with perforated bottoms, pour on boiling water, and then give it time to drain through; but if the liquid is then boiled, its most essential and distinctive quality is evaporated and lost, although not to as great an extent as in the most unphilosophical American method.

